# Determinants of Men's Involvement in Maternity Care in Dodoma Region, Central Tanzania

**DOI:** 10.1155/2019/7637124

**Published:** 2019-06-02

**Authors:** Nyasiro S. Gibore, Mangi J. Ezekiel, Alfred Meremo, Mariam J. Munyogwa, Stephen M. Kibusi

**Affiliations:** ^1^School of Nursing, College of Health Sciences, University of Dodoma, P.O. Box, 395, Dodoma, Tanzania; ^2^School of Public Health, Muhimbili University of Health and Allied Sciences, P.O. Box 65001, Dar es Salaam, Tanzania; ^3^School of Medicine, College of Health Sciences, University of Dodoma, P.O. Box 395, Dodoma, Tanzania

## Abstract

**Background:**

Men's involvement in maternity care is recognized as a key strategy in improving maternal health and accelerating reduction of maternal mortality. This study investigated the factors determining men's involvement in maternity care in Dodoma Region, Central Tanzania.

**Methods:**

This cross-sectional survey used multistage sampling in four districts of Dodoma Region to select 966 married men participants aged 18 years and above. Data were collected using a structured questionnaire. Multivariate logistic regression analysis was carried out in SPSS version 21.0 to measure the determinants of men's involvement in maternity care.

**Results:**

The study found that only 1 in 5 men were involved in maternity care of their partners. Factors found to determine men's involvement in maternity care were having >4 children (AOR=1.658, 95%CI=1.134 to 2.422), urban area of residence (AOR=0.510, 95%CI=0.354 to 0.735), waiting time >1 hour at the health care facility (AOR=0.685, 95%CI=0.479 to 0.978), limited access to information (AOR=0.491, 95%CI=0.322 to 0.747), and limited spousal communication (AOR=0.3, 95%CI=0.155 to 0.327).

**Conclusions:**

Long waiting time to receive the service and limited access to information regarding men's involvement are associated with low men's involvement in maternity care. Male friendly maternity care should recognize men's preferences on timely access to services and provide them with relevant information on their roles in maternity care. Spousal communication is important; mothers must be empowered with relevant information to communicate to their male partners regarding fertility preferences and maternity care in general.

## 1. Introduction

The need to include men in maternal health care was as a result of the 1994 International Conference on Population and Development (ICPD) held in Cairo, Egypt, which urged that special efforts should be made to emphasize men's shared responsibility and promote their active involvement in responsible parenthood; sexual and reproductive behavior including family planning; and prenatal, maternal and child health (ICPD, POA: paragraph 4.27) [1]. In most families around the world, men tend to be responsible for important choices relating to the allocation of household resources and care seeking behavior that directly impact the health of women and newborns [2]. Several studies have revealed the benefits of involving men in maternity care, such as increased access to antenatal care services which leads to increased likelihood of delivery by skilled birth attendants [3] and increased access to modern family planning methods [4], as well as addressing gender-related barriers to access maternal health services [5].

Despite the fact that there has been increasing recognition of the need to include men in maternal health care since the mid-1990s, actual progress towards engaging men in maternity care has been slow in most developing countries [2]. For instance, the study conducted in Uganda reported low male involvement in the prevention of mother to child transmission of HIV [6] and the study in Nigeria reported low male involvement in maternity care [7]. Similarly, different studies conducted in Tanzania have reported a low male involvement in different activities of maternity care [8] and low rate of male participation in prevention of mother to child transmission (PMTCT) and in voluntary counseling and testing [9, 10].

Studies have reported several factors contributing to low male involvement in maternity care. The common reported include cultural beliefs, gender norms, financial constraints, health care providers attitude, community perceptions, infrastructure, crowded birthing suites, and lack of privacy for laboring and birthing families in the delivery rooms, which inhibit male partners' presence [6, 7, 11–15]. Other factors reported by other scholars were communication among couples and waiting time for antenatal services in the health care facility. The study done by Shahjahan found that lack of communication among couples on reproductive health matters creates difficulty for men to understand reproductive problems of women [16]. Decision making and care seeking is a process that requires good communication between partners. Lack of communication among couples often resulted in men being unaware of women's health care seeking intentions which resulted in men's limited understanding of reproductive needs of women [17]. One study suggested that social relationships may determine people's capacity to manage their sexual and reproductive health, with important implications not only for their health but also for other life choices [18]. Some scholar reported that many negative conditions can be avoidable if the pregnant woman gets social and psychological support, not only from high quality maternal and child health care but also from their social network, especially their partners [19, 20]. Long waiting time for antenatal services in the health care facilities was also reported as a contributing factor for low men's involvement in maternity care. The study conducted in different parts of African countries reported that both women and men had concern that they spent long times in clinics to receive antenatal care services and male partners cannot just waste their valuable times in clinics for hours waiting for services [6, 12, 17, 21, 22].

In Tanzania, the issue of men's involvement in maternity care gained impetus after recognition that couples' joint HIV counseling and testing during antenatal care would help improve prevention of mother to child transmission (PMTCT) outcomes and that male involvement in PMCTC services would help to achieve the Millennium Development Goals of combating HIV/AIDS, reducing child mortality and improving maternal health [23, 24]. In view of the importance of male involvement in PMTCT, Tanzania Ministry of Health and Social Welfare included male involvement policy strategy in its PMTCT guidelines, which states that “all maternal and child health (MCH) facilities should put strategies in place to ensure male involvement in antenatal, natal, and postnatal care as well as child health monitoring” [23]. Since then health service providers and some nongovernmental organizations in Tanzania have been implementing different strategies to promote male involvement in maternity services, such as offering incentives to couples who attend ANC clinics together and sensitisation and education programmes for couples to prepare them for maternity care [25].

Despite having these strategies in place, there are limited studies regarding the determinant of men's involvement in maternity care in Tanzania. This study seeks to establish the factors determining men's involvement in maternity care in Dodoma Region. Findings from this study could inform the design of intervention, strategies towards improving men's involvement in maternity care.

## 2. Materials and Methods

### 2.1. Setting

The study was conducted in Dodoma Region. The area was selected because it is the capital of the country and a fast growing region with a cultural diversity befitting the examination of male involvement in maternity care. Therefore, it was assumed that studying the determinants of men's involvement in maternity care in Dodoma Region would provide a broad picture of the study findings from different cultures in Tanzania. Dodoma Region has seven districts; four districts were randomly selected to be involved in the study namely: Kondoa, Kongwa, Chamwino, and Dodoma Municipality. Dodoma Region is located in central part of Tanzania, with a population of 2,083,588 people and population density of 50 people per square kilometers. It covers an area of 41,310 square kilometers [26]. Male population accounts for 48.7% of the total population. The annual population growth rate is 2.1% with a sex ratio of 95 males to 100 females [26]. The region's health care service structure is made up of seven hospitals, 32 health centers, and 269 dispensaries, most of which provide reproductive and child health services [26].

### 2.2. Study Design, Participants, and Sampling Procedure

This study employed a descriptive cross-sectional survey using quantitative research approach. It involved married men aged 18 years and above, who resided with their spouses together in the same household, whose partner had a child aged two years or below, and whose partners had second pregnancy and above at the time of data collection and was willing to participate in the study. The study was conducted between November 2016 and June 2017.

Sample size was estimated using the Kish Leslie's formula based on the following assumptions: 95% confidence level, 39.2% estimated prevalence (findings from a previous study [8]), a 5% margin of error and a design effect assumed to be 2.5 to cater for intracluster variability; the sample was further increased by 20% to account for nonresponse or recording error [27]. Therefore, the estimated total sample size was 1,099 respondents. A three-stage cluster sampling strategy was used to select a representative sample from the four districts. First, all wards in the four districts were listed and then two wards in each district were randomly selected using the ballot method, which made a total of eight wards. In the second stage, all streets and villages in the selected wards were listed and then two streets in Dodoma Municipality and two villages in each three districts (Kondoa, Kongwa, and Chamwino) were randomly selected. In stage three, list of houses was obtained and then proportionate samples were drawn from each district. A systematic sampling technique with the starting point obtained using a table of random numbers was used to select the houses. In cases where more than one household was found in a house, one household was selected by using a single one-time ballot. In the households if a man had more than one partner with a child born within the past two years, the interview was conducted based on the information from the youngest child. Eligible men in the sampled household were approached to participate in the study.

A structured, interviewer-administered questionnaire containing open and close-ended questions was used to collect data. This data collection tool was adapted from the previous works [6, 28]. The adaptation of the questionnaire was based on the aim and objectives of our study, literature review and relevant local factors related to the research question. The questionnaire was divided into three parts. The first part captured information on household social demographic variables. The second part assessed the level of men's involvement in maternity care during antenatal, natal, and postnatal periods. The third part assessed the determinants of men's involvement in maternity care.

Prior to data collection, the questionnaire was pretested in Bahi district, which has similar characteristics as the districts selected for study. The questionnaire was modified accordingly before being used in the study. It was administered by eight male research assistants who had recently graduated from medical school and were trained by principal investigator for 3 days before the start of data collection. The interviews were conducted in Swahili language.

### 2.3. Measurement of Variables

Prior to actual analysis of the data, the data were cleaned, validated, and analyzed using SPSS version 21.0. The dependent variable (men's involvement in maternity care) was constructed as a single variable to obtain the involvement index using twelve dichotomized (yes/no) variables. The study assessed four activities and each activity had three variables as follows: (1) accompanies partner to antenatal, natal, and postnatal care, (2) provides physical and emotional support to his partner during antenatal, natal, and postnatal periods, (3) is involved in joint planning for antenatal care, place of delivery, and postnatal care, and (4) discusses maternal health issues with her health care providers during antenatal, natal, and postnatal periods. Factor analysis was performed to obtain male involvement index. The purpose was to measure how much each variable contributes to the outcome variable (male involvement). All twelve variables were subjected in the principal component analysis. In the first analysis four components with eigenvalues (variance) greater than one were extracted. According to “Kaiser's rule” only those components with eigenvalues greater than one should be retained [29]. Based on Kaiser's rule the study decided to retain the first component because it had greater eigenvalue (variance) than other components. In the first component the variables that had correlation coefficients score of less than 0.3 were excluded in the second analysis. Correlation coefficient (*r*) must be 0.30 or greater since anything lower would suggest a really weak relationship between the variables [30]. In this study six variables were found to have a correlation coefficient less than 0.3 which indicated a weak relationship with the outcome variable. The variables that had weak relationship were provides physical support during postnatal period, provides physical support during natal period, is involved in joint planning for place of delivery, is involved in joint planning for postnatal care, provides physical support during antenatal period, and accompanies partner to delivery of the child. These variables were excluded in the second factor analysis. The second factor analysis was performed with the remaining six variables. Two components with eigenvalues greater than one were extracted. Based on the same rule “Kaiser's rule” the first component was retained because it had greater eigenvalue than the second component and this first component was the one used to obtain men's involvement index score.

After obtaining the scores of each respondent, the median, minimum, and maximum values of the scores were calculated as follows: mean was 0.7137965, median was 0.3460358, minimum score was -1.94712, and maximum score was 1.15192. To obtain the scores in percent the percentile was set as 0-50 low involvement and 51-100 as high involvement. Based on the median, mean, and maximum values the percentile was calculated and categorized as -1.94712 to less than 0.7137965 as low involvement and above 0.7137966 to 1.15192 as high involvement. Lastly the categories were coded as “0” for low involvement “1” for high involvement and the frequency of overall involvement score was obtained.

### 2.4. Independent Variables

Preventive cultural norms/taboo was measured by asking the respondents if there are any cultural norms or taboos which prevent them from accompanying their partners to the health care services and they were required to respond if it is yes/no. The variable attitude was measured by asking the respondents the following question: how do you find the attitude of health workers towards men who accompany their partners to hospital to seek care? The question had two options: (1) they attend to us very well and friendly and (2) they are unfriendly. Those who answered option one had a positive attitude and number two were regarded as negative attitude. Access to information was measured by asking the following question: have you ever heard or been told that men are supposed to attend at antenatal care services with their partners? (yes/no). Time spent while waiting for ANC service was measured by asking the following question: how long on average do you spend in the health facility when you accompany your partner for ANC service? The responses obtained were summarized into two categories (less than or equal to one hour/ more than one hour). Spousal communication was measured by asking the respondents the following question: do you discuss or ask your partner any issues related to her pregnancy and delivery? Respondents were required to answer if it is yes/no.

### 2.5. Data Analysis

The data was entered, cleaned, validated, and analyzed using Statistical Package for Social Sciences (SPSS Version 21.0). Variables were tabulated using frequencies and percentages. The* Chi-square test *was used for testing the significance of association between categorical variables. A bivariate analysis was carried out and crude odds ratios (ORs) for each variable were calculated. All variables that were significantly associated with men's involvement in maternity care were included in a multivariate logistic regression analysis in order to determine their independent effects in maternity care. The Adjusted ORs and their corresponding 95% Confidence Interval (CI) were obtained. The level of significance was set at P < 0.05.

## 3. Results

### 3.1. Sociodemographic Characteristics of Respondents

Of the 1,099 participants sampled for the study, 966 interviews were completed, giving a response rate of 88%. The nonrespondents (12%) were not available at home for the interview after repeated visits at the time of data collection. The sociodemographic characteristics of study participants are shown in Table 1. The age of respondents ranged from 18 to 70 years (mean ± standard deviation = 40.0±11.8 years). Over 62% of the respondents were between 25 and 44 years. The number of children per respondent ranged from one to twenty-two children (mean ± standard deviation = 3.9±11.8 children). Over 67% of respondents were having one to four children. The majority (74.1%) of respondents were Christian and most (74.5%) of whom belonged to the Gogo and Rangi ethnic groups. Majority (70.4%) of respondents were peasants (agricultural and agropastoral). More than two-thirds (77.5%) of respondents had a primary education. Majority (91.5%) of respondents were in monogamous relationship.

### 3.2. Level of Men's Involvement in Maternity Care

The level of male involvement in maternity care was assessed using the variables shown in Table 2. Only 15 (1.6%) of the 966 men had accompanied their partners to the health care facility during delivery (natal) period, but more than half of them (63.4%) and (64.1%) accompanied their partners to ANC and postnatal care, respectively. Most of respondents (77.3%), (82.2%), and (82.2%) provided physical support to their partners during ANC, natal and postnatal care, respectively. Similarly planning for seeking care during ANC, natal and postnatal care was made by (89.0%), (88.0%), and (90.2%) of respondents, respectively. Less than a quarter (23.5%), (19.7%), and (21.0%) of respondents made discussion on maternal health issues with their partners' health care providers during ANC, natal, and postnatal care, respectively.

The level of men's involvement in three periods of maternity care is shown in Table 3. The high level of involvement is seen in antenatal (53.9%) and postnatal (59.3%) periods of care while the natal period of care had high proportion (84.2%) of low level of involvement than other periods.

**Figure 1:**
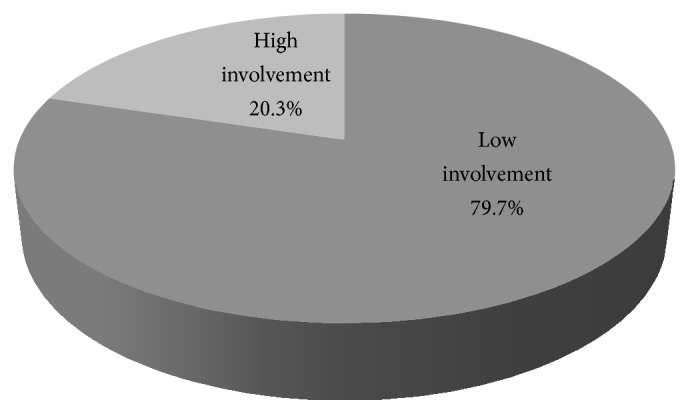
Overall level of men's involvement in maternity care.

The overall level of involvement was obtained by performing principal component analysis. Only 196 (20.3%) of the 966 respondents had a high male involvement index while 770 (79.3%) had low involvement index, Figure 1.

### 3.3. Factors Associated with the Level of Men's Involvement in Maternity Care

Table 4 shows the factors associated with the level of men's involvement in maternity care. The study found that number of children, place of residence, waiting time at the health care facility, access to information on men's involvement in maternal health care services, and spousal communication on matters related to reproductive health were the significant factors in men's involvement in maternity care.

Table 5 shows the results of logistic regression analysis to measure the determinants of men's involvement in maternity care. The variables that showed association with men's involvement in maternity care in cross tabulation (chi-square) were subjected into logistic regression analysis. In order to adjust for the effect of confounders, the variables, number of children, place of residence, waiting time, access to information, and spousal communication were entered simultaneously in the logistic regression model. After adjustment for the effect of confounders, the variables number of children, access to information, spousal communication, place of residence, and waiting time at the health facility remained significantly associated with high men's involvement in maternity care. Men who had more than four children were more likely to have high men's involvement index than their counterparts (AOR=1.658, 95%CI=1.134 to 2.422); men who had no access to information regarding their involvement in maternity care were 2 times less likely to have high men's involvement index than men reported to have access to information (AOR=0.491, 95%CI=0.322 to 0.747). Men who had no communication with their partners on issues related to maternity care were 3 times less likely to have high men's involvement index than men who had communication with their partners (AOR=0.3, 95%CI=0.155 to 0.327). However, it was surprising to note that men who resided in urban area were 2 times less likely to have high men's involvement index than men resided in rural area (AOR=0.510, 95%CI=0.354 to 0.735). Men who reported to spend more than one hour waiting for the services at the health care facility were less likely to have men's involvement index than their counterparts (AOR=0.685, 95%CI=0.479 to 0.978).

## 4. Discussion

This study found that only 1 in 5 men were involved in different activities of maternity care. This level of involvement is lower than what is reported from other studies in Tanzania, Uganda, and Kenya. The study conducted in Pwani Region Tanzania showed that male involvement in different activities of maternity care was 39.2% [8]. The study in Uganda found that 26.0% of male participants had high involvement [6] and the study conducted in Nigeria reported that 32.1% of male were involved in maternity care [7]. The differences observed in these study findings could be due to difference in study setting, sample size, geographic location, and ineffectiveness of implementation of safe motherhood program by the different national health systems. Health care providers and program implementers should take appropriate action to advocate and encourage men's involvement in maternity care. During ANC attendance pregnant women and their partners are given health education together. This may result in a greater outcome on maternal health involvement behavior as compared to when women receive this education alone [31].

This study revealed some determinants of men's involvement in maternity care. These include number of children, place of residence, waiting time at the health care facility, access to information on men's involvement in maternity care, and spousal communication. Our finding that men with more children had high involvement suggests that fertility preference may determine men's involvement in maternity care. In Tanzania, fertility preference is 5.2 children per woman [32]; it is likely that men who have and wish to have more children feel more pressured to be involved in maternity care. On the other hand, there could be more concerns with the health of the mother as the number of pregnancies and babies thereof increases. Another possible explanation could be due to the familiarities with the health system. In Africa men are not familiarized with the health system compared to women. The fact that they have once attended the health facility for previous children deliveries conditions they are more likely to come back when they are invited compared to the new fathers. Our finding differs with the study conducted in north western Tanzania which found that having less than two children was associated with higher male attendance at reproductive health service [33]. Similarly, other studies reported that having or intention to have more children was associated with lower male participation in maternity care [34–36]. This finding could imply that fertility preferences could be bespoke if both partners attend ANC during maternity periods. Thus, health education about family planning which is given during antenatal and postnatal visits should not only encourage child spacing but also the number of children that the couple can manage to take care of. A qualitative study is needed in order to understand the perspective of men on fertility preference and their involvement in maternity care.

Men who resided in rural areas were more likely to have high involvement in maternity care compared to their urban counterparts. One possible explanation of this finding could be that in the rural areas where the health care facilities are located far from the people coupled with unreliable transport and limited finance to pay for transport, walking or cycling was the main means of transport to the health care facility for most of respondents. So, this necessitated men to accompany their partners for fear of their safety while they are walking or travelling a long way to the health care facility. By doing so they found themselves at the health care facility. Alternative explanation for this finding could also be a successful outreach programmes in rural areas, including the Mwanzo Bora Nutrition Project, Safe Motherhood Initiative Programmes, and Mother and Child Health Information System. The existence of these health projects may have created awareness and influence their involvement in maternity care. Therefore, this provides opportunity for health care providers, voluntary agencies, and Ministry of Health to identify and train peer educators who will act as change agents to inform and encourage other men to be involved in maternity care.

Another factor that was found to determine high men's involvement in maternity care was waiting time at the health care facility. Men who reported to spend less than one hour to receive services at the health care facility were more likely to be involved in maternity care compared to their counterparts. This finding is similar to Mullick and Wanjiru who reported that long waiting time at the health facility was one of the reasons for low rate of male accompanying their partners to maternal health services [12]. Ditekemena et al. in Democratic Republic of Congo and Nkuoh et al. in Cameroon had similar findings [21, 22]. In order to achieve the waiting time of less than one hour at a health care center as per WHO standards, a situation analysis to understand the context in which health care providers work in is necessary, as the lack of time, lack of inputs, and large patient loads can inhibit health care providers motivation and ability to support and promote male involvement subject, as male involvement in maternity care is a new approach in the country that was introduced with the Prevention of Mother To Child Transmission (PMTCT) program.

Men who reported to have access to information regarding men's involvement in maternity care were more likely to have high involvement in maternity care compared to their counterparts. This finding suggests that access to credible source of information may lead men to make informed decisions and consequently result in positive behavior change. The alternative explanation to this finding is that the effectiveness of health education given to men who attended at ANC may have motivated their involvement. Access to and use of the information obtained during ANC visits can influence men to utilize maternity health services with their partners and be able to manage pregnancy complications as early as possible. This finding is consistent with other studies in Uganda and Zambia which found that men who had heard information regarding PMTCT services were more likely to be involved in PMTCT activities than their counterparts. Additionally, men who obtained health information from health workers were more likely to accompany their partners at ANC [6, 36, 37]. The health sector is by default expected to play as the main source for maternal health information for men. Thus, providing suitable and appropriate information to men is important for their decisions on involvement in maternity care.

This study also found that communication among partners increased the likelihood of high men involvement in maternity care. This finding implies that good communication among partners could result in positive support from male partner regarding maternal health issues, as it has been emphasized in the ICPD that it is important to enhance communication between couples on sexual and reproductive health since men play a key role in decision making on health care matters [38]. The finding of our study is similar to other studies in Nepal and Kenya where good couple communication was found to improve men's involvement behavior [39–41]. This finding also supports evidence from a systematic review by Ditekemena et al. who reported that poor communication between men and their partners was associated with poor men's involvement [21]. Thus, interspousal communication among partners on matters related to maternity care is very crucial in achieving health benefits of the mother and child. Communication among couples and access to information on the existence of service for both partners enhance men's involvement in maternity care eventually resulting in improved maternal health and reduced child mortality and morbidity and accelerating the achievement of the sustainable development goal 3 (three).

## 5. Strengths and Weaknesses of the Study

The strength of this study is based on its large sample size, sampling procedures, and higher response rate, which assures that the findings represent majority of the study population and promotes the results confidence. There are two main limitations in this study. First, men's involvement in maternity care is an extremely complex topic of discussion, which cannot be measured by a single variable. This study assessed only four variables in three periods of maternity care. However, the combination of other variables such as partners' communication regarding family planning, men providing adequate nutrition, ability to identify danger signs in their partners, arranging transport and financial support for their partners during delivery and emergencies could improve the accuracy of the measure of men's involvement. Secondly, since the study design was cross-sectional, there are issues of recall bias and yet pregnancy issues may not mean as much importance to the male partners as they do to the female. This could have been tackled by cross validation of the men's responses by women's opinions through interviews. The design also limits the study from making any causal inferences in relation to the main outcome and independent variables.

## 6. Conclusions 

The level of men's involvement in maternity care in Dodoma was low. Access to information on men's involvement in maternity care, spousal communication, place of residence, number of children ever born and waiting time at the health care facility were the determinants of men's involvement in maternity care. These findings provide a helpful reference for targeting men's involvement interventions in the future.

However, the long times spent by men waiting for the services when accompanying their partners have a negative impact which is reflected in men's limited involvement. Since the long waiting times and access to men's involvement information are attributed to the issues to do with health care facilities, this study concludes that, without adequate, well trained, responsible, and conscientious health care providers who are sensitive to the feelings of the people they serve according to service seekers' situation, it will be difficult to change positively men's involvement subject in practice. It is, therefore, recommended that the Ministry of Health should plan appropriate interventions to fill the gap that causes time lag and negative impact on men's response to maternity care. The study also recommends qualitative study to be undertaken which will highlight the barriers for men's involvement in maternity care. This should go hand in hand with increasing community awareness on men's involvement in maternity care at all levels of health care delivery.

## Figures and Tables

**Table 1:** Sociodemographic characteristics of respondents (N = 966).

Variable	Category	N (%)
Age	15 - 24	56 (5.8)
	25-34	294 (30.4)
	35-44	309 (32.0)
	≥ 45	307 (31.8)
*Occupation*	Employed	80 (8.3)
	Agricultural	653 (67.3)
	Agro pastoral	30 (3.1)
	Business	144 (14.9)
	Casual laborers	59 (6.1)
Education level	No education	136 (14.1)
	Primary education	749 (77.5)
	Secondary education	62 (6.4)
	Tertiary education	19 (2.0)
Ethnicity	Gogo	515 (53.3)
	Rangi	205 (21.2)
	Sandawe	4 (0.4)
	Kaguru	86 (8.9)
	Hehe	21 (2.2)
	Others (Fipa, Mbulu, Ngoni)	135 (14.0)
Religion	Christian	716 (74.1)
	Muslim	250 (25.9)
Marriage type	Arranged Marriage	56 (5.8)
	Chosen each other	910 (94.2)
Marriage relationship	Monogamous	884 (91.5)
	Polygamous	82 (9.5)
Access to Information	Yes	829 (85.8)
	No	137 (14.2)
Number of children	1-2	327 (33.9)
	3-4	322 (33.3)
	≥ 5	317 (32.8)
Distance to health facility	≤ 5 kilometers	854 (85.0)
	≥ 5 kilometers	110 (15.0)

**Table 2:** Proportion of respondents involved in the four activities used to measure the level of men's in maternity care.

Level of involvement	Frequency (%)
*During antenatal period*	
Accompanies partner to the health care facility	612 (63.4)
Provides physical support to his partner	747 (77.3)
Involved in planning for seeking care	860 (89.0)
Discusses maternal health issues with her health care providers	227 (23.5)

*During delivery (natal) period*	
Accompanies partner to the health care facility	15 (1.6)
Provides physical support to his partner	794 (82.2)
Involved in planning for seeking care	850 (88.0)
Discusses maternal health issues with her health care providers	190 (19.7)

*During postnatal period*	
Accompanies partner to the health care facility	619 (64.1)
Provides physical support to his partner	794 (82.2)
Involved in planning for seeking care	871 (90.2)
Discusses maternal health issues with her health care providers	203 (21.0)

**Table 3:** Level of men's involvement in maternity care during antenatal, natal, and postnatal care.

Period of care	Level of involvement (N=966)	
	High	Low
	n (%)	n (%)
Antenatal	521 (53.9)	445 (46.1)
Natal	153 (14.9)	813 (84.2)
Postnatal	573 (59.3)	393 (40.7)

**Table 4:** Factors associated with the level of men's involvement in maternity care (N=966).

Variables	Level of involvement		Chi-square	p-value
	Low (770)	High (196)		
	n (%)	n (%)		
*Age (years)*				
18-30	170 (76.2)	53 (23.8)	2.167	0.145
>30	600 (80.8)	143 (19.2)		
*Education level*				
Not educated	58 (80.6)	14 (19.4)	0.034	0.853
Educated	712 (79.6)	182 (20.4)		
*Ethnicity*				
Gogo	413 (80.2)	102 (19.8)	0.160	0.689
Others (rangi, sandawe, etc.)	357 (79.2)	94 (20.8)		
*Number of children*				
1-4	496 (77.4)	145 (22.6)	6.401	*∗∗*
>4	274 (84.3)	51 (15.7)		
*Occupation*				
Employed	210 (75.8)	67 (24.2)	3.648	0.056
Not employed	560 (81.3)	129 (18.7)		
*Religion*				
Christian	571 (79.7)	145 (20.3)	0.003	0.960
Muslim	199 (79.6)	51 (20.4)		
*Place of residence*				
Rural	333 (84.1)	63 (15.9)	7.964	*∗∗*
Urban	437 (76.7)	133 (23.3)		
*Attitude of health care worker*				
Positive	642 (80.5)	156 (19.5)	1.558	0.212
Negative	128 (76.2)	40 (23.8)		
*Waiting time*				
≤ One hour	294 (83.1)	60 (16.9)	3.856	*∗*
>One hour	476 (77.8)	136 (22.2)		
*Distance to the health unit*				
≤ 5km	676 (79.1)	179 (20.9)	1.919	0.166
>5km	94 (84.7)	17 (15.3)		
*Access to information *				
Yes	681 (82.2)	147 (17.8)	23.052	*∗∗∗*
No	89 (64.5)	49 (35.5)		
*Spousal communication*				
Yes	646 (85.0)	114 (15.0)	61.662	*∗∗∗*
No	124 (60.2)	82 (39.8)		

*∗*Statistically significant at *∗*p < 0.05, *∗∗*p < 0.01, *∗∗∗*p < 0.001.

**Table 5:** Determinants of men's involvement in maternity care (N=966).

Variables	OR	95%C.I.		P value	AOR	95%C.I.		P value
		Lower	upper			Lower	upper	
*Number of children*								
1-4	1				1			
>4	1.571	1.105	2.232	0.012	1.658	1.134	2.422	**∗** **∗**
*Place of residence*								
Rural	1				1			
Urban	0.622	0.446	0.866	0.005	0.510	0.354	0.735	**∗** **∗** **∗**
*Waiting time*								
≤ One hour	1				1			
>One hour	0.714	0.510	1.000	0.050	0.685	0.479	0.978	**∗**
*Access to information on male involvement*								
Access	1				1			
No access	0.392	0.265	0.580	0.001	0.491	0.322	0.747	**∗** **∗** **∗**
*Spousal communication*								
Yes	1				1			
No	0.267	0.189	0.376	0.001	0.225	0.155	0.327	**∗** **∗** **∗**

*∗*Statistically significant at *∗*p < 0.05, *∗∗*p < 0.01, *∗∗∗*p < 0.001, OR=Odds Ratio, AOR= Adjusted Odds Ratio.

## Data Availability

The datasets used and/or analyzed during the current study are available from the corresponding author on reasonable request.

## List of Abbreviations

AIDS: Acquired Immune Deficiency Syndrome
ANC: Antenatal Care
AOR: Adjusted Odd Ratio
CL: Confidence Interval
HIV: Human Immune Deficiency Virus
ICPD: International Conference on Population and Development
MCH: Maternal and Child Health
MMR: Maternal Mortality Ratio
PMTCT: Prevention of Mother To Child Transmissions
POA: Program of Action
SPSS: Statistical Package for Social Sciences
WHO: World Health Organization.
